# The use of Xbox Kinect™ in a Paediatric Burns Unit

**DOI:** 10.4102/sajp.v74i1.429

**Published:** 2018-04-09

**Authors:** Eleonora I. Lozano, Joanne L. Potterton

**Affiliations:** 1 Department of Physiotherapy, University of the Witwatersrand, South Africa; 2 Chris Hani Baragwanath Academic Hospital, Johannesburg, South Africa

## Abstract

**Background:**

The popularity of video game use in burns rehabilitation has grown because, in addition to facilitating maintenance of range of motion (ROM), the virtual imaging characteristics of these games provide distraction from pain.

**Objectives:**

The effect of using Xbox Kinect™ as an adjunct to physiotherapy in a Paediatric Burns Unit (PBU) has not been established. This study aimed to investigate the effect of using the Xbox Kinect™ on outcomes of children in the PBU at Chris Hani Baragwanath Academic Hospital.

**Methods:**

This non-equivalent, post-test only control group study took place over a period of 14 months. The control group received standard physiotherapy management and the experimental group received standard physiotherapy management and additional Xbox Kinect™. Outcome measures were ROM, Activities Scale for Kids (ASK©p) and a modified Wong-Baker FACES^®^ enjoyment rating scale. Outcomes were assessed at discharge and then 1 week post-discharge at the usual follow-up appointment.

**Results:**

Sixty-six children participated in this study. More than 50% of the burns were because of hot water, followed by flame burns (30%) and electrical burns (12%). The addition of Xbox Kinect™ was effective in achieving higher active ROM (AROM) between discharge and follow-up (*p* < 0.01). Fun and enjoyment (*p* < 0.01) was found to be significantly higher in the children who participated in Xbox Kinect™. Total body surface area (TBSA%) (*p* = 0.03), age (*p* = 0.05) and AROM (*p* = 0.04) were significantly associated with ASK©p scores.

**Conclusion:**

The use of the Xbox Kinect™ has been shown to be a beneficial and useful adjunct to burns rehabilitation in this paediatric burns population.

**Clinical implications:**

Currently, there is limited information and research on interventions for children with burns in South Africa. The addition of Xbox Kinect™ to standard physiotherapy in-patient care was both enjoyable and effective and should be considered where funding is available.

## Introduction

Burns are a predominant cause of paediatric injuries and contribute to more than 90% of paediatric deaths owing to injury primarily in low- and middle-income countries (Wesson et al. 2013). Hot water and scald burns are the most common causes of burns in children younger than 5 years of age, with flame burns more common in older children (Lesher et al. 2011).

Schmitt et al. (2011) describe physiotherapy as an essential, sometimes painful, component of burns rehabilitation. Early and aggressive physiotherapy can facilitate and counteract the decreased range of motion (ROM), and most importantly prevent severe contractures and potential disability that can develop secondary to burns and associated skin grafting. Thus, burn rehabilitation is fundamental to improving functional outcomes and decreasing long-term disability (Schmitt et al. 2011). Pain and anxiety are critical factors that may considerably influence the course of recovery. Acute pain is exacerbated by anxiety (Yohannan et al. 2012).

Parry et al. (2015) describe the aims of exercise as being to preserve and restore movement and improve function; these aims can be achieved by the use of age-appropriate play activities, functional training and tasks, muscle strengthening and conditioning exercises alongside passive stretches and active ROM (AROM) exercises. Children, as opposed to adults, will often require more motivating strategies and treatment programmes as they are fearful of moving their burnt limbs and refuse to do so owing to pain, anxiety and fear (Parry et al. 2015).

There has been a growing trend and interest in finding cost-effective therapeutic approaches for rehabilitation. Part of this trend is the use of commercially accessible video games like the Nintendo^®^ Wii™, the Playstation™ II Eye Toy and the Microsoft^®^ XBox 360 Kinect™ as part of rehabilitation to enhance physiotherapy management.

The popularity of video game use in burns rehabilitation has grown because, in addition to facilitating and encouraging ROM in an effort to prevent the formation of joint contractures, the virtual imaging characteristics of these games provide the advantageous benefit of distraction from pain (Parry et al. 2015). Video games provide an effective, efficient and enjoyable method of training and are a helpful addition to rehabilitation (Kho et al. 2012).

Microsoft’s Xbox Kinect™ is a video game device that uses an infrared camera and sensor to detect a user’s movement. Unlike other gaming devices, there is no need for a special controller or hand-held device. The player’s movement is captured in real time with immediate visual and auditory feedback being provided to the player (Sin & Lee 2013). The Microsoft Xbox Kinect™ video games are promising rehabilitation and treatment options, because they involve total body movements achieved in a motivating and fun manner (Levac et al. 2015).

The aim of this study was to investigate the effect of using the Xbox Kinect™ on discharge outcomes and early activity levels of children in the Paediatric Burns Unit (PBU) at Chris Hani Baragwanath Academic Hospital (CHBAH). This study compared the discharge outcomes of two groups of children, where one group received standard physiotherapy management and the other group received standard physiotherapy management plus Xbox Kinect™ during their admission stay in the PBU.

## Method

This study took place at the Johnson and Johnson PBU at CHBAH in Soweto. Soweto is the largest township in the Gauteng province in South Africa and has a population of 1.3 million people, which accounts for 40% of Johannesburg’s population (Frith 2011). The PBU admits children from birth to 12 years of age.

This study was a non-equivalent, post-test only control group design which took place over a period of 14 months. It was not possible to randomise the children admitted to the PBU, as they all stay in the same room and are exposed to the same environment; so, the treatment offered to the children at any time-point needs to be the same.

In 2014, the PBU had 581 new burns admissions of which 112 were aged more than 5 years. The highest number of admissions was seen during the winter months of June–October 2014. Using the central limit theory, a minimum of 30 children was required in each group to avoid a type 1 error in this study.

Children were included in the study if they were aged 5–12 years, willing to participate, their parents had consented, and they were admitted with burns of any percentage, severity and cause. Children were excluded if they:

- had previous and current medical problems that may have impaired aspects of joint range, for example: haemophilia, previous contractures, septic arthritis or cerebral palsy
- had been re-admitted.

Range of motion of affected joints was assessed with the use of a goniometer. Despite limitations shown with goniometry measurement, Gajdosik and Bohannon (1987) noted that the goniometer is a valid clinical assessment tool. The passive ROM (PROM) and AROM were measured for all affected joints. Normal values for ROM of joints were compared and a percentage of AROM and PROM calculated compared to that joint’s specific ROM (Moroz 2013). For example, shoulder flexion full ROM is 180°, if the AROM was 90° the AROM% was 50% (90/180) and if the PROM was 180° the PROM% was 100%.

The Activities Scale for Kids (ASK©) has been identified as an outcome measure examining the functional ability of a child with musculoskeletal physical disability (Christakou & Laiou 2014). The ASK© is a 30-item self-report questionnaire for children aged 5–15 years (Christakou & Laiou 2014; Young 2009). Young (2009) states that ‘the ASK© may be used to assess a child’s status at a single point in time or to monitor changes associated with time or therapeutic interventions’.

The ASK© has nine subdomains which address personal care, dressing, eating and drinking, miscellaneous, locomotion, stairs, play, transfers and standing skills. There are also eight additional information items which look at the use of assistive devices and the degree of assistance the child requires (Christakou & Laiou 2014). The ASK© has two versions: the ASK©-capability (ASK©c) and the ASK©-performance (ASK©p). The ASK©c measures physical function in a hypothetical or ideal setting of what the child ‘could do’ and the ASK©p, which is more commonly used, assesses what the child ‘does do’ (Christakou & Laiou 2014; Piscione, Davis & Young 2014).

The ASK© has been designed to be completed by the child and not through clinical observation, as it has been shown that children are able to report and have an understanding of their own physical disability (Piscione et al. 2014). The ASK© has undergone thorough testing showing that it is a clinically useful assessment tool with sound reliability and validity for children aged between 5 and 15 years (Young 2009). It measures what the child ‘did do’. It was developed as a need to evaluate therapeutic effectiveness (Christakou & Laiou 2014).

A modified Wong-Baker FACES^®^ enjoyment rating scale with points ranging from zero to five, with zero indicative of ‘no fun at all’ represented by a sad face and five indicative of ‘extremely fun’ represented by a very happy face was used to assess the enjoyment of the intervention (Wong-Baker FACES^®^ 2016).

Children who were eligible to participate were identified with daily screening of the unit by physiotherapy staff working in the unit and they and their parents were invited to participate in the study. Their parents were approached during visiting times and informed about the study and provided with information sheets explaining the study and its purpose. Nursing staff and the physiotherapy assistant working in the unit who were aware of the study assisted with translations where necessary.

Each child was assigned a study code, and all information and study-related data were collected on a data collection form by the first author, E.I.L. The control group was the first group of children recruited to the study. These children all received standard physiotherapy management carried out by the physiotherapy staff working in the unit. The intervention group was the second group of children recruited to the study, who all received standard physiotherapy management plus the Xbox Kinect™ intervention.

The Xbox Kinect™ intervention was only started once all the children in the control group had been discharged. Analysis of the data was done only after both groups had undergone their respective interventions by the first author.

Once discharged from the unit, a discharge summary form was filled out for each child by the first author. ROM measurements were done on the day of discharge from the unit and at the follow-up clinic. Active ROM and passive ROM were measured once the wounds had been checked by the doctors, without limitation of any bandages or dressings, and adequate analgesia was provided. Active ROM was measured first followed by PROM measurement of the affected joint. Only one ROM measurement was recorded and used.

Demographic information, burn depth and severity, surgical management, the number of physiotherapy treatment sessions and Xbox Kinect™ sessions were all recorded on the discharge summary form and the data collection form. Each child was also asked to rate enjoyment of the physiotherapy sessions using the modified Wong-Baker FACES^®^ enjoyment rating scale.

Follow-up of the recruited children took place at the PBU Outpatients clinic 1 week after discharge. Children discharged from the PBU are required to attend this clinic for wound inspection from the medical staff and functional assessment by the therapists working in the PBU. The ASK©p was done with the child and parent before wound exposure and ROM assessment was done once the wounds and dressings were exposed and changed.

All data and information were confidentially stored in a file which was only accessible to the first author. Electronic data were also stored on a personal computer and were only accessible to the first author. In the PBU at CHBAH, physiotherapy is commenced on the first day of hospital admission and recommenced on the fifth day post-skin grafting until discharge. Treatment sessions are between 30 and 45 min taking place at least 1–2 times daily (Monday to Friday).

The control group children received standard physiotherapy management which consisted of daily goal-orientated therapy aimed to maintain normal ROM and muscle strength, prevent contracture formation and facilitate normal activities of daily living (ADLs) and activity based on the protocols set in the PBU. Parents and caregivers are also educated, demonstrated to, and taught specific exercises for their child during their stay as part of the supervised home exercise programme needed once discharged.

On the days when the children had dressing changes, passive mobilisation and stretches were done in the dressing room under ketamine analgesia as set by protocols in the PBU. These treatment sessions were carried out by the physiotherapy staff working in the unit and took place in the mornings.

Each child in the intervention group received standard physiotherapy management and Xbox Kinect™ sessions. The Xbox Kinect™ sessions took place in the afternoons in the PBU therapy gym and children were given the choice between Kinect Sports™ and Dance Central 3™ games to engage in. Kinect Sports™ offers different sporting activities such as boxing, soccer, track-and-field games, while the Dance Central 3™ game offers a variety of sing-a-long and dancing games that the children were required to mirror and replicate.

A minimum of twice weekly Xbox Kinect™ sessions of 15–30 min each were conducted. The activities could be done either sitting or standing approximately 1.5 m–2 m from the screen and the infrared camera sensor. These treatment sessions were carried out by the physiotherapy staff and students working in the unit, all of whom had received training with the Xbox Kinect™ prior to commencing the study.

Descriptive statistics, for example mean, median, standard deviation (SD) and interquartile range (IQR), were used to describe the demographic characteristics of the population, as well as the length of stay (LoS) and number of physiotherapy management sessions for each group.

The level of significance was set at *p* ≤ 0.05. Paired *t*-tests were used to analyse the ROM and the ASK©p scores and activity domain data of the total sample and the two groups at discharge and at follow-up. A Spearman’s correlation was used to establish any association between the two groups for the ROM results. To establish the relationship and association found between total body surface area (TBSA%) and ASK©p scores, we used an F-test and then a linear regression model. A Mann–Whitney U test was used to analyse the fun and enjoyment data from the FACES score.

### Ethical consideration

Prior to commencing data collection for this study, ethical clearance was obtained from the Human Research Ethics Committee (Medical) of the University of the Witwatersrand (No. M150727). Permission was granted by CHBAH to conduct the study in the hospital’s PBU.

## Results

### Demographics

**FIGURE 1:**
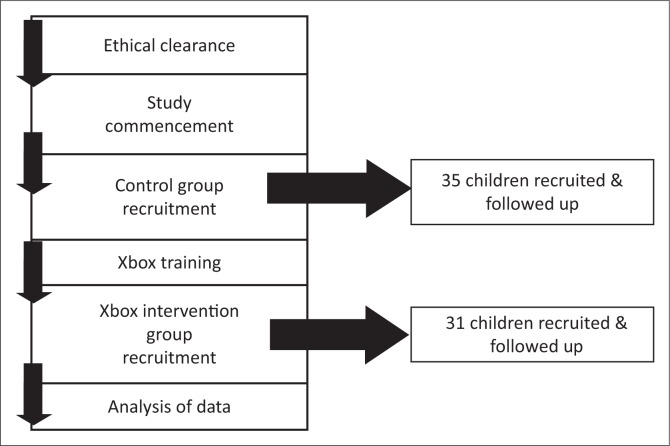
Flow chart summarising recruitment of study population.

Seventy children were approached for recruitment into the study, of whom 66 assented to participate and parental consent was obtained. Thirty-five children were in the control group and 31 in the Xbox intervention group, as depicted in the flow chart in Figure 1.

No significant difference was found between groups regarding demographic characteristics. The median age was 7 (range 5–9) years, and 55% of the participants were male. The majority of burns were hot water burns accounting for more than 50% of the admissions, followed by flame burns (30%) and electrical burns (12%). Clinical data showing the TBSA%, burn depth, burn severity and cause of burn are described in Table 1.

**TABLE 1:** Demographic data showing total body surface area percentage, burn depth, burn severity and cause of burn for study population (*n* = 66).

Total *n* = 66	Control group *n* = 35	Xbox intervention group *n* = 31	*p*
**Median burn TBSA% (IQR)**	8 (4.5–16)	10 (8–15)	0.58
**Burn depth (*n*/%)** Superficial Superficial partial Deep partial Full	3 (8.6%) 20 (57.1%) 8 (22.9%) 4 (11.4%)	4 (12.9%) 19 (61.3%) 4 (12.9%) 4 (12.9%)	0.74
**Burn severity (*n*/%**) Minor Moderate–severe Severe	21 (6.0%) 11 (31.4%) 3 (8.6%)	15 (48.4%) 16 (51.6%) 0 (0.0%)	0.78
**Cause of burn (*n*/%**) Hot water Flame Electrical Other	18 (51.4%) 11 (31.4%) 5 (14.3%) 1 (2.9%)	17 (54.8%) 9 (29.0%) 3 (9.7%) 2 (6.4%)	0.84

IQR, interquartile range; TBSA, total body surface area.

With regard to the overall TBSA% in the study population, 9% had burns that were superficial–partial in depth, seen as a minor burn injury. Forty per cent were seen to have moderate–severe injury and three children were considered to have severe, major burns > 30% TBSA. We observed a greater proportion of injury involving the lower limbs (23.1%) and upper limbs (21.1%), followed by injury involving the trunk (11.4%), buttocks and genitalia (7.5%) and head and neck regions (6.8%).

In Table 2, the number of surgeries, split skin grafts and Biobrane^®^ dressing for each group is demonstrated which was not found to be significantly different. There was no significant difference in the median LoS in days between the two groups with 10 (range 7–18) days for the control group and 16 (range 10–29) days for the Xbox intervention group. There was no difference in the median number of physiotherapy sessions between the two groups (*p* = 0.23), with 7.5 (range 5–12) days for the control group and 11 (range 10–29) days for the Xbox intervention group.

**TABLE 2:** The number of surgeries, length of stay and the number of physiotherapy treatment sessions for the study population (*n* = 66).

Total *n* = 66	Control group *n* = 35	Xbox intervention group *n* = 31	*p*
Surgeries Split skin graft Biobrane^®^	15 14 2	11 15 5	0.25
Mean LoS days (IQR)	10 (7–18)	16 (10–29)	0.21
Median number of physiotherapy treatment sessions (IQR)	7.5 (5–12)	11 (7–20)	0.23

LoS, length of stay; IQR, interquartile range.

### Range of motion

For analysis of ROM between the two groups, only areas that were burnt were analysed, as unaffected areas and joints had full AROM. As seen in Table 3, there was no significant difference between PROM scores at discharge and at follow-up (*p* > 0.5). Five children in our study population developed contractures, three from the control group and two from the Xbox intervention group.

**TABLE 3:** The percentage of passive range of motion and active range of motion for the total sample and the two groups at discharge and at follow-up (*n* = 66).

Treatment group	Total *n* = 66	Control group *n* = 35	Xbox intervention group *n* = 31
Average percentage of normal PROM at discharge	96.4	93.5	98.6
Average percentage of normal PROM at follow-up	95.7	93.2	98.2
Difference from discharge to follow-up (*p*)	0.7 (0.5)	0.3 (0.7)	0.4 (0.3)
Average percentage of normal AROM at discharge	62.5	58.9	66.2
Average percentage of normal AROM at follow-up	79.6	74.2	85.0
Difference from discharge to follow-up (*p*)	17.1 (*p* < 0.01)	15.3 (*p* = 0.06)	18.8 (*p* < 0.01)

PROM, passive range of motion; AROM, active range of motion.

Despite the treatment methods used in this study of aggressive and early ROM exercises and the use of splinting, two of the children in this study developed unilateral axillary shoulder contractures, while the remaining three developed bilateral contractures involving the hip or knee joints.

There was a significant difference in the overall AROM between discharge (62.5%) and follow-up (79.6%) for the total sample (*p* < 0.01) and a significant difference was also seen between the percentage of AROM from discharge to follow-up in the Xbox Kinect™ intervention group (*p* < 0.01). The AROM at discharge in the Xbox intervention group was 66.2% compared to 58.9% in the control group, while the AROM at follow-up was 85% in the Xbox intervention group compared to 74.2% in the control group.

### Activities Scale for Kids©p

No significant difference was found for the median ASK©p scores for the two groups at follow-up. We found that TBSA% was associated with ASK©p scores (*p* = 0.03); thus, the higher the burn percentage the lower the ASK©p scores, as indicated in Table 4.

**TABLE 4:** Total body surface area percentage associated with Activities Scale for Kids©p scores.

Total *n* = 66	*p*
TBSA %	0.03*
Age	0.24
Treatment group	0.87
Sex	0.29

TBSA, total body surface area.

* , indicates significant *p*-value.

A moderate correlation between the ASK©p score and overall ROM was found (*r* = 0.45; *p* < 0.01).

Following a linear regression, we also found that age (−1.89; *p* = 0.05) and AROM (0.22; *p* = 0.04) were significantly associated with ASK©p scores; thus, a young child or a child with reduced AROM would have lower ASK©p scores. This is shown in Table 5.

**TABLE 5:** Age and active range of motion as predictors of Activities Scale for Kids©p scores.

Total *n* = 66	Coefficient	*p*
Sex	−2.86	0.46
Treatment group	0.10	0.98
Age	−1.89	0.05*
Burn depth	0.58	0.46
AROM	0.22	0.04*

AROM, active range of motion.

* , indicates significant *p*-value.

### Fun and enjoyment

More fun and enjoyment was experienced in the Xbox intervention group (*p* < 0.01), the median FACES score for the control group and the Xbox intervention group were 3 (range 3–4) and 5 (range 4–5), respectively. This highlights the fun and enjoyment factor the Xbox Kinect™ offered as part of therapy and as an adjunct to standard physiotherapy management in this study.

## Discussion

This study aimed to investigate the effect of using the Xbox Kinect™ as an adjunct to physiotherapy on discharge outcomes of children in the PBU at CHBAH as this has not been established.

The significant difference in overall AROM between discharge and follow-up in the two groups is in keeping with the work done by Parry et al. (2015), in which when active therapy and supervised home exercise programmes are implemented improvements in ROM are shown. The largest improvements of ROM are seen when exercise is initiated early in the rehabilitation programme (Parry et al. 2015).

The significant difference found in the percentage of AROM from discharge to follow-up in the Xbox intervention group compared to the non-significant difference in the control group highlights the advantages the Xbox Kinect™ has in providing a more amusing and comfortable option as part of the burns rehabilitation process as described by Mobini, Behzadipour and Foumani (2014). By allowing the children to be more engaged in the Xbox Kinect™ experience and games, they were distracted and thus experienced less pain as previously described by Parker et al. (2015). This decline in pain assists in reducing the fear associated with movement these children experience and assists in improvements related to activity and ultimately age-appropriate play and ADLs.

The ASK©p was used as an assessment measure of performance once the child was discharged and was back in their familial home environment. Analysis of the domains and which areas the children scored lower highlights the potential for treatment planning and goal setting the clinician and family can address to improve the overall scores and functioning of the child.

We found that TBSA, age and AROM were associated with functional outcomes, in comparison to Tyack and Ziviani (2003) who conducted a longitudinal cohort design study investigating what factors influence the functional outcomes of children aged 5–14 years 6 months post-burn injury. The burn injury factors associated with TBSA, number of surgical procedures and type and cause of burn did not have a significant impact on functional outcomes in their study. Age of the child was found to significantly affect the variance of the functional outcome; thus, the younger the child the better the functional outcomes as seen in this study (Tyack & Ziviani 2003).

The children who took part in the Xbox intervention group had much higher scores and fun and enjoyment values compared to fun and enjoyment values of the control group. These children come from low socioeconomic and underprivileged backgrounds, and may have found this technology to be motivating as they would not have had access to it in their daily lives.

Of the 66 children analysed, the majority were male (55%) which is similar to previous burns studies done by Jugmohan et al. (2016) and Albertyn, Bickler and Rode (2006) which reported that males more commonly had burn injuries. Three similarities and trends were noted between the five children who developed the contractures; these were of the male gender, the injury was as a result of flame burns and was a full-thickness burn depth injury. These three trends and similarities could help to identify factors that contribute to the increased risk of contracture formation among burn injuries.

Jeschke and Herndon (2014) explain that with a full-thickness injury, the wound will not heal by itself. Should the wound heal via grafting and coverage, it will heal alongside hypertrophic scarring thus increasing the risk of contracture formation (Jeschke & Herndon 2014). Overall, these five children were seen to have major and severe burn injury. All of them required grafting, which again highlights the increased formation of contractures explained by Webb et al. (2011) where contractures are most likely associated with larger TBSA and increased depth of the burn, as well as a larger area requiring grafting (Webb et al. 2011).

A limitation of the study is that a single blinded randomised control trial would have been the optimal design for this study; however, owing to the way the unit is run, this was not an ethical option. In addition, owing to these constraints, it was impossible for the researcher to be blinded to the group the children were in. It is possible that this introduced an element of bias to the study. It is also possible that as the groups did not run concurrently, small differences in seasonal variation of burn presentation could have occurred. This study was a preliminary exploratory study where all children with all types of burns were included for analysis; however, this did make interpretation of the results difficult as the clinical presentation varied widely and so results cannot be generalised. Future studies could concentrate on moderate severity burns so that the sample is more homogenous (Voon et al. 2016). A further limitation of this study was that the modified Wong-Baker^®^ FACES enjoyment scale that was used had not been validated. A validated measure to assess enjoyment should be used in future studies.

## Conclusion

This study was the first study done in South Africa involving video game technology during physiotherapy management within the paediatric burns population. The use of the Xbox Kinect™ as seen in this study has been shown to be a useful adjunct to burns rehabilitation in the paediatric burns population.

It was also shown to be fun, enjoyable and highly motivating to help these children with burns improve function and to be distracted from pain.
